# Why do older adults decline participation in research? Results from two deprescribing clinical trials

**DOI:** 10.1186/s13063-023-07506-7

**Published:** 2023-07-18

**Authors:** Thomas E. Strayer, Emily K. Hollingsworth, Avantika S. Shah, Eduard E. Vasilevskis, Sandra F. Simmons, Amanda S. Mixon

**Affiliations:** 1 Center for Quality Aging, Vanderbilt University Medical Center, 2147 Belcourt Ave., Nashville, TN 37212 USA; 2 Division of Geriatrics, Vanderbilt University Medical Center, 2147 Belcourt Ave., Nashville, TN 37212 USA; 3 Department of Health Policy and Management, Johns Hopkins Bloomberg School of Public Health, Baltimore, MD USA; 4 Geriatric Research Education and Clinical Center, VA Tennessee Valley Healthcare System, 1310 24Th Ave. S, Nashville, TN 37212 USA; 5 Section of Hospital Medicine, Division of General Internal Medicine & Public Health, Vanderbilt University Medical Center, 2525 West End Ave. Suite 450, Nashville, TN 37203 USA

**Keywords:** Polypharmacy, Deprescribing, Patient engagement

## Abstract

**Background:**

Heterogenous older adult populations are underrepresented in clinical trials, and their participation is necessary for interventions that directly target them. The purpose of this study was to evaluate reasons why hospitalized older adults declined participation in two deprescribing clinical trials.

**Methods:**

We report enrollment data from two deprescribing trials, Shed-MEDS (non-Veterans) and VA DROP (Veterans). For both trials, inclusion criteria required participants to be hospitalized, age 50 or older, English-speaking, and taking five or more home medications. Eligible patients were approached for enrollment while hospitalized. When an eligible patient or surrogate declined participation, the reason(s) were recorded and subsequently analyzed inductively to develop themes, and a chi-square test was used for comparison (of themes between Veterans and non-Veterans).

**Results:**

Across both trials, 1226 patients (545 non-Veterans and 681 Veterans) declined enrollment and provided reasons, which were condensed into three themes: (1) feeling overwhelmed by their current health status, (2) lack of interest or mistrust of research, and (3) hesitancy to participate in a deprescribing study. A greater proportion of Veterans expressed a lack of interest or mistrust in research (42% vs 26%, chi-square value = 36.72, *p* < .001), whereas a greater proportion of non-Veterans expressed feeling overwhelmed by their current health status (54% vs 35%, chi-square value = 42.8 *p* < 0.001). Across both trials, similar proportion of patients expressed hesitancy to participate in a deprescribing study, with no significant difference between Veterans and non-Veterans (23% and 21%).

**Conclusions:**

Understanding the reasons older adults decline participation can inform future strategies to engage this multimorbid population.

## Introduction

In 2019, the National Institutes of Health released the “Inclusion across the Lifespan” policy aimed to guide clinical research to be inclusive of all ages, including older adults [10]. Numerous barriers have been cited as potentially contributing to the lack of representation of older adults in clinical trials such as poor health status, accessibility issues, social and cultural barriers, decision-making capacity, age discrimination, and lack of family support or agreement. Furthermore, these barriers may be intensified during an acute event such as hospitalization [5, 6, 16, 24].

It is important for older adults with diverse health status to participate in clinical trials addressing clinical issues in which older age is a substantial risk factor, such as polypharmacy. Polypharmacy is associated with a number of adverse outcomes including geriatric syndromes such as urinary incontinence and falls, medication errors, cognitive impairment, and healthcare utilization [1, 4, 8, 12–14, 20]. The issue of polypharmacy in older adults has recently led to the development and implementation of deprescribing interventions that focus on the reduction of unnecessary or potentially harmful medications [15, 16, 18, 19]. It is critical for deprescribing studies to ensure that enrolled participants represent the target population of older adults, which is heterogenous. There is little data related to factors that may contribute to the recruitment of older adults for studies to evaluate novel drug treatments or interventions to improve medication regimens. The purpose of this study was to evaluate the reasons older adults declined participation in two patient-centered deprescribing intervention trials initiated in the hospital setting (one trial among Veterans and the other trial among non-Veterans). The overarching goal of this analysis was to understand the concerns of both Veterans and non-Veterans who declined participation to inform future recruitment strategies that engage older adult populations in clinical trials.

## Methods

We report enrollment data from two randomized controlled trials to evaluate a patient-centered deprescribing intervention to reduce unnecessary medications for hospitalized patients discharged to skilled nursing facilities (SNF). In both trials, deprescribing recommendations were made based on the entirety of each participant’s medication list, rather than being focused on a specific medication class, and incorporated both patient preferences and their treatment teams’ agreement. The Shed-MEDS trial (NCT02979353) was conducted from 2015 to 2020 at an academic medical center, and the VA DROP trial (NCT03722017) was conducted from 2019 to 2023 at one Veterans Affairs hospital, with both enrollment sites located in the middle Tennessee area. Both trials required patients to be aged 50 or older, English-speaking, and taking five or more medications prior to hospitalization. Additionally, those who were receiving long-term care or hospice care were excluded from both trials, and those with housing instability were excluded from the Shed-MEDS trial but included in VA DROP due to having an assigned social worker to assist the research team in contacting the Veteran after hospital discharge.

Our university-affiliated institutional review board (IRB) provided a HIPAA waiver for eligibility screening to be conducted through electronic chart review, and then eligible patients were approached by trained research study personnel (nurse practitioners, pharmacists, or non-clinical research assistants) for enrollment during hospitalization. For patients unable to provide self-consent, a surrogate was approached for enrollment. Both studies required the patient (or their surrogate, if unable to self-consent) to complete standardized assessments lasting 1 h at enrollment and up to three times via telephone following hospital and SNF discharge. The assessments measured medication-related outcomes such as adherence, functional health status, attitudes toward deprescribing, and geriatric syndromes (e.g., incontinence, falls). The intervention consisted of a pharmacist or nurse practitioner led comprehensive medication review and required patient/surrogate to agree upon deprescribing recommendations. Deprescribing actions were initiated in the hospital for both studies and for Shed-MEDS, continued past hospital discharge. The complete protocol for the Shed-MEDS trial has been published previously [19, 22, 23]. All study procedures within both trials were approved by affiliated institutional review boards (IRB) and data safety monitoring boards (DSMBs).

There were only a few differences between the two trials with Shed-MEDS including one follow-up home visit and modest compensation for participation; otherwise, study procedures and assessments were the same across the two trials. While the trial protocols were similar, eligible participants were expected to differ given that Shed-MEDS was conducted at an academic medical center with a high proportion of insured, urban/suburban patients, whereas VA DROP was conducted at a Veterans’ Administration facility with a high proportion of male and potentially rural patients.

## Data collection and analysis

Eligible patients and/or surrogates were provided with study information primarily in-person by trained study personnel until COVID-19 restrictions in 2020 necessitated remote options such as electronic consent (Shed-MEDS) or telephone consent (VA DROP). Telephone consent required a second study team member to serve as a witness to the consent procedure. When an eligible patient or surrogate declined participation, study personnel attempted to elicit their reason(s) with structured prompts (e.g., “would you be willing to share the reason you are not interested in participating in this study?”) then documented their responses using standardized forms to track study enrollment. Initial content coding for each documented reason followed by content analysis to synthesize, integrate, and organize the data was performed by three of the co-authors (TS, EH, and AS) inductively to develop common categories and themes and a codebook with definitions for each theme, with discussion and agreement from all study team members [3]. As both studies continued recruitment, new reasons for declining enrollment were added to the content analysis and codebook and discussed, as necessary, to reach agreement. The IBM SPSS Version 28 was used for data management and conducting descriptive statistics (*IBM SPSS Statistics for Windows, Version 27.0*, n.d.). Chi-square tests were used to compare themes between the two study populations (Veterans versus non-Veterans).

## Results

Across both studies, a total of 2279 eligible patients were approached by study personnel and 1330 declined to enroll, of which 1226 (92.2%) provided at least one reason for declining participation. Of those 1226 patients, 545 (45%) non-Veterans declined participation in Shed-MEDS, and 681 (55%) Veterans declined participation in VA DROP. While patients and/or surrogates provided a total of 1510 reasons for declining study participation (because participants could provide more than one reason for declining), we present analysis for the primary or first reason provided. The research teams identified 14 reasons (subthemes) provided by patients and surrogates, which were subsequently recoded into three main themes (Table 1).

**Table 1:** Overarching theme and subthemes for reasons for declining participation in two deprescribing randomized controlled trials

Theme	Subtheme	Example quotes
**Lack of interest or mistrust of research**	• I’m not interested in participating in any research • Unwilling to sign any study-related documents • General mistrust of health care systems	**Shed-MEDS:** Surrogate reported “No, [I’m not interested] in participating in any research” and would not allow study team to describe research **VA DROP:** Patient reported “I am not interested [in any study]”as soon as study team entered room
**Overwhelmed by current health status**	• I’m too overwhelmed (by my medical condition, decisions I need to make right now, etc.,) • I need more time to think or consult with others (family, doctor, etc.,) before agreeing to participate • Surrogate uncomfortable making the decision(s) on patient’s behalf • Time (or timing) to participate is not convenient or feasible • Patient unwilling to enroll via remote consent (during COVID-19 pandemic restrictions)	**Shed-MEDS:** Patient reported “I do not want anything else on my plate [right now].” **VA DROP:** Surrogate reported “patient is not in their right mind, and I am overwhelmed because I am having to do everything [for the patient].”
**Hesitancy to participate in deprescribing study**	• I don’t think I meet study criteria • My doctor has already reduced the number of medications I take • I only want my doctor changing my medications • I feel comfortable with my medicines and don’t want to make any changes • Previous negative medication change experience • Patient does not wish to have risks of being in the study	**Shed-MEDS:** Patient reported “my new doctors at [the hospital] are getting my medications in line and I don’t want anyone else touching them” **VA DROP:** Surrogate reported “every time that the medications are changed, [the patient] goes downhill, and [the patient] is doing well right-now.”

**Fig. 1:**
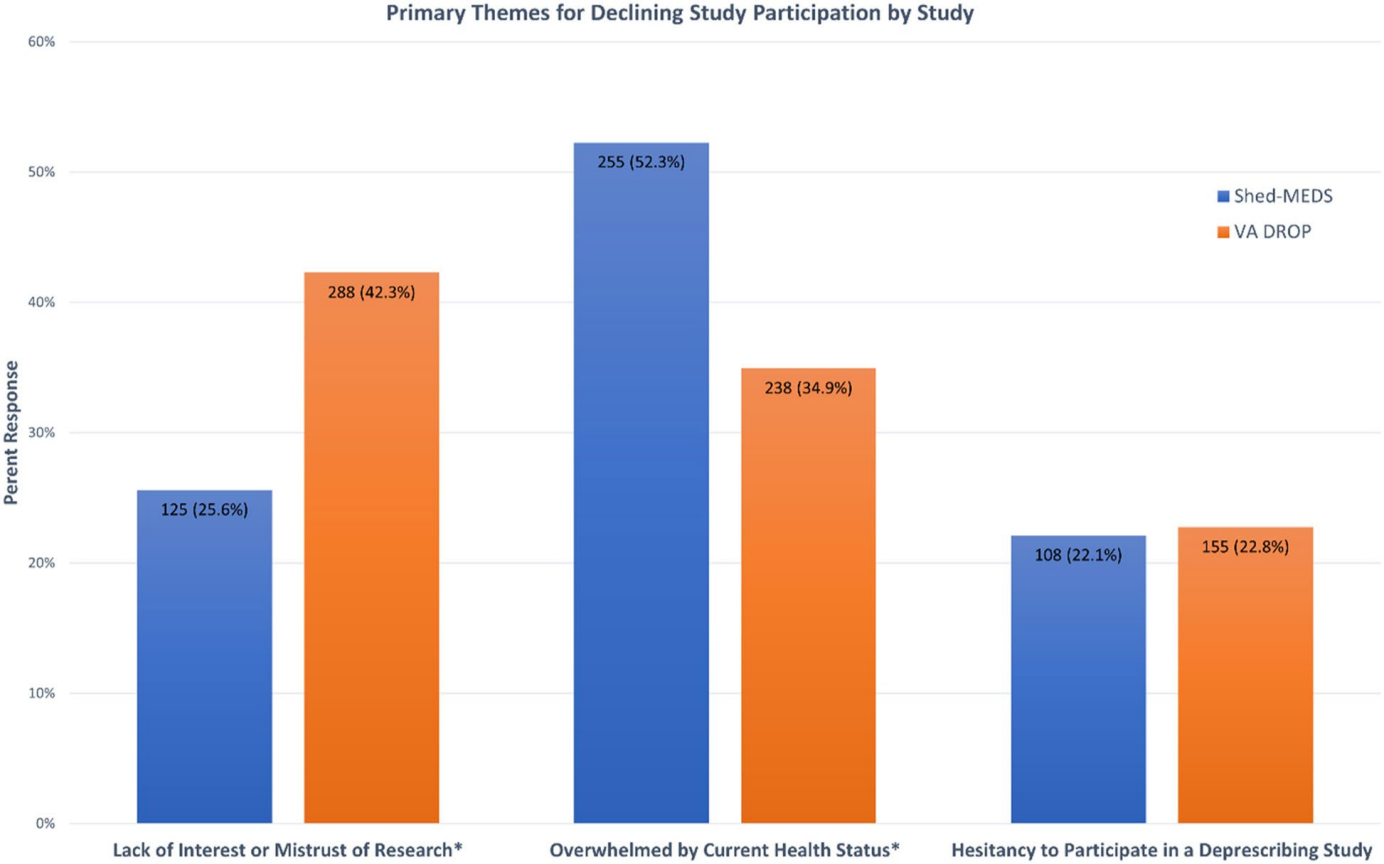
Primary themes for declining study participation. *Indicates a statistical difference (p<0.001) between study groups

Figure 1 shows the proportion of eligible patients within each theme by study, and Table 1 provides the 14 subthemes and examples of patient (or surrogate) responses for each theme. Across both studies, the broader themes provided by eligible patients for declining enrollment were “feeling overwhelmed by their current health status” (43%), “lack of interest or mistrust of research” (35%), and “hesitancy to participate in a deprescribing study” (22%). Hesitancy to participate in a deprescribing study included patients’ expressed comfort with their current medication regimen, desire for medication changes to be made only by their prescribing providers, and/or a prior negative experience with a medication change.

As shown in Fig. 1, there were significant differences between the two studies for the reasons for declining enrollment. A significantly greater proportion of non-Veterans reported “feeling overwhelmed by their current health status” as the primary reason (Fig. 1, 54% vs 35%, chi-square = 42.8 *p* < 0.001), whereas a greater proportion of eligible Veterans for VA DROP reported a “lack of interest or mistrust of research” (Fig. 1, 42% vs 26%, chi-square = 36.72, *p* < 0.001). A comparable proportion of eligible patients in both studies declined to enroll due to an expressed “hesitancy to participate in a deprescribing study” (Fig. 1, 21% and 23% for Shed-MEDS and VA DROP) which indicates a potential enrollment bias in both trials toward patients more willing to stop or reduce their medications.

## Discussion

This descriptive study explored the reasons hospitalized older adults declined participation in two deprescribing trials. We identified three main themes: feeling overwhelmed by their current health status, a lack of interest or mistrust in research, and hesitation toward the deprescribing intervention. Older hospitalized patients’ reports of “feeling overwhelmed by their current health status” is consistent with prior studies demonstrating that health status impacts the ability or desire of older adults to participate in clinical research [5, 20]. Because hospitalized older adults transitioning to SNF were the target population for both trials, their rationale for not participating due to “feeling overwhelmed” may have been influenced by multiple factors including changes in their health status that lead to their hospitalization and possible feelings of uncertainty about SNF placement following hospital discharge [5]. This theme also included a subtheme of an expressed need for more time to have conversations with family members and/or providers about possible study enrollment, and the hospitalization period limited the time available. These findings suggest that the clinical setting wherein patients are approached for study enrollment (e.g., hospital setting versus SNF or outpatient settings) may influence their willingness to enroll.

The reported “lack of interest or mistrust in research” suggests a need to improve older adults’ understanding of clinical trial research and the potential benefits of participation. This effort also should address common myths or biases about research participation (e.g., not wanting to be a “guinea pig”). In this study, a greater proportion of Veterans expressed this concern relative to non-Veterans as a reason for declining enrollment, although it remained common in both groups. One potential way to address this barrier might be to consider the training and professional credentials of the study personnel responsible for enrollment [2]. Although we did not collect data related to the type of study personnel and related enrollment rates, it is plausible that a clinician might instill more confidence in a patient and contribute to better enrollment rates compared to a non-clinical research assistant for health-related intervention trials. Researchers also can engage stakeholders, such as those who have undergone deprescribing, to develop recruitment materials and strategies that meet the needs of the target population and address common concerns or misperceptions. While some materials have been created to educate older adults on trial participation, such as the ROAR Toolkit, their primary audience is healthy, community dwelling (non-hospitalized) older adults [17]. It is also unclear if these resources are being widely publicized and therefore may be underutilized.

Lastly, a significant proportion of both groups declined participation due to hesitancy toward the deprescribing intervention. This category included patients reporting comfort with their current medications, despite meeting clinical criteria for polypharmacy, and/or the desire for medication changes to be made only by their prescribing providers, or a prior negative experience with a medication change. Overall, concerns about medication changes represented approximately 20% of the reasons for declining enrollment, which suggests a potential enrollment bias toward patients more willing to deprescribe in both clinical trials, which is important to acknowledge when examining intervention effects. Hesitancy toward enrolling in a deprescribing intervention might also indicate a lack of awareness or understanding of the risk of polypharmacy or patient-centered deprescribing interventions. This finding suggests that it may be informative for researchers to document reasons for declining enrollment during recruitment for clinical intervention trials as the potential for enrollment bias (i.e., those more aware of and/or willing to comply with an intervention) is relevant to the evaluation of many types of behavioral health interventions.

Representativeness in the population might be better achieved through tailoring study procedures based on the intervention, setting, and target sub-population. Additionally, studies should include multiple recruitment strategies with resources and personnel acceptable to the target population. Currently, the US Deprescribing Research Network formally engages patient and community stakeholders to increase overall awareness on the topic and elicit feedback on deprescribing study designs (US Deprescribing Network, n.d.).

### Limitations

This descriptive study has a few notable limitations. First, in the absence of consent, we were unable to collect additional demographic or clinical characteristic data beyond our study eligibility criteria for those who declined enrollment. Because this content analysis to determine the reasons for declining participation was not part of our original study aims, our HIPAA waiver for screening purposes was limited to only those data necessary to determine study eligibility as defined by the inclusion and exclusion criteria for the trials. Thus, we were unable to assess demographic data (e.g., age, gender, ethnicity/race) for those who declined participation. Such data would be informative to determine the influence of other patient characteristics on willingness to enroll as well as their reasons for declining enrollment. Additionally, not all those who declined enrollment provided a reason, and this study was not designed to include in-depth qualitative interviews of eligible patients to ascertain all possible reasons for declining enrollment; thus, there could be other factors that influenced eligible patients’ decision beyond those captured in these data. Lastly, we could not conduct comparisons between patients and surrogates because these groups were not consistently differentiated in the absence of consent. Other studies have suggested that surrogates may have different attitudes toward deprescribing relative to patients [7]. Thus, deprescribing trials targeting surrogates and/or family caregivers, such as those for patients with Alzheimer’s disease and related dementias, may encounter different reasons for declining participation.

## Conclusion

The purpose of this study was to report the reasons older adults (or their surrogates) declined participation in two deprescribing clinical trials. Given the growing evidence that deprescribing can be conducted safely and effectively, pragmatic trial approaches with alternative study designs and consenting procedures tailored to the population’s specific concerns may promote inclusion of a broader eligible patient population [11, 18]. Additionally, adaptive enrollment strategies developed in conjunction with relevant stakeholders could help researchers achieve higher enrollment rates in clinical trials. As the implementation of deprescribing interventions continue, the need to identify patient populations likely to benefit and their reasons for declining participation remains important. This study highlights the need to consider older adults’ potential reasons for declining enrollment in the early phases of study design to allow descriptive data to be appropriately captured for all eligible persons. These efforts will improve our understanding of patient barriers to participation and inform the design of interventions with a broader reach and impact.

## Data Availability

The raw datasets generated and/or analyzed during the current study are not publicly available but are available from the corresponding author on reasonable request if approved by the Vanderbilt University Institutional Review Board and completion of a formal data sharing agreement. Data from the VA DROP study can be available upon reasonable request.

## Abbreviation

SNF: Skilled nursing facility
